# Model Predictive Control with a PSO Modelling Approach for Position Control of a Compliant Ankle Rehabilitation Robot

**DOI:** 10.3390/biomimetics11050349

**Published:** 2026-05-16

**Authors:** Dexter Felix Brown, Sheng Quan Xie, Yiliu Tu

**Affiliations:** 1 School of Electronic and Electrical Engineering, University of Leeds, Leeds LS2 9JT, UK; el18dfb@leeds.ac.uk; 2 Modern College of Engineering, Henan Normal University, Xinxiang 453007, China; tuyiliu@htu.edu.cn

**Keywords:** control systems, machine intelligence, modelling, optimisation algorithms, predictive algorithms, rehabilitation robots

## Abstract

The Compliant Ankle Rehabilitation Robot (CARR) is actuated by four Pneumatic Artificial Muscles (PAMs). These actuators mimic biological muscles, making them highly applicable in robotic systems designed to apply guiding motion to a human joint, but have complex nonlinear dynamic properties making accurate tracking control difficult. This paper presents intelligent modelling and control methods to improve the CARR’s function in a rehabilitation setting. Using the Particle Swarm Optimisation (PSO) algorithm, dynamic models of the actuators are calculated. Two model setups are proposed, a single model and dual model. Model Predictive Control (MPC) was then implemented using these models and experimentally compared with Proportional Integral Derivative (PID) and Iterative Learning Control (ILC). The results showed that PID control was less accurate than the developed control schemes, with evidence of significant chattering, as well as both over- and undershooting the setpoint. ILC performed accurately, but the required learning period and some evidence of overfitting impacted the overall performance. Single-model MPC had low error values on the X axis of the CARR and maintained the most consistent 0 displacement when an axis was intended to stay motionless. Dual-model MPC had the lowest error values on the Y axis, the smoothest motion with the least chattering, and the best performance when both axes were in motion, but showed evidence of overshooting. Based on these results, both MPC implementations have proven to be successful and suitable for future work, and the PSO modelling method is able to produce suitably accurate models for the application.

## 1. Introduction

The field of rehabilitation robotics has seen a rapid increases in both research and applications [1], with the global market expected to grow by $5 billion by 2032 [2]. With the ever-increasing interest and usage of these robots in both research and clinical environments, it is becoming increasingly important that they are safe, reliable, and are held to a high standard of functionality. To this end, significant research in the field concerns intelligent algorithms and control structures [3,4], with many designs mentioning user comfort and safety as a measure of success alongside more commonplace measures like motion and force tracking accuracy [5,6]. The use of compliant actuators and structures in the design of rehabilitation robots is an effective way to improve patient comfort and safety by allowing for some freedom of motion during usage, as well as increasing the transparency of the robot by reducing interaction torques between the robot and patient. Flexible pneumatic actuators, such as PAMs, allow for compliant actuation with sufficient torque capabilities to apply proper force output for rehabilitation exercises, making them a suitable and increasingly popular choice for actuation in the field. PAMs are designed to mimic the dynamic properties of biological muscles, incorporating flexibility and elasticity, and using similar operating principles in contracting based on internal pressure. This allows for mechanical motion inherently similar to that experienced by human joints, which is especially helpful when designing robotic systems to apply guiding motion and forces to a person. However, their elastic construction and actuation method relying on pressurised air makes them more complicated to control than traditional actuators, often leading to less accurate motion and force tracking. Intelligent control strategies are generally more suitable than traditional control for these dynamic actuators, as their unique properties can be better accounted for. There exists great variety in the literature concerning the specific intelligent algorithms used in control systems, even within the field of rehabilitation robotics. Learning control methods such as ILC allow for improved performance over time using system feedback and measured error values, making them applicable to systems with repeated, predictable input sequences [7]. However, this necessarily means that the initial motion accuracy is poor before the algorithm has had a chance to learn, and adjusting the learning parameters to speed up learning can lead to instability. Adaptive controllers are especially suitable for rehabilitation as they allow for dynamically adjusted control parameters based on certain feedback or measurements, helping improve both motion accuracy and comfort for users [8]. These algorithms can be applied to many different control schemes but will ultimately increase the complexity and computational load. The use of fuzzy logic is also common in control architecture, as it allows for heuristic decision-making and easy grouping of continuous data [9]. However, some accuracy is inevitably lost in the fuzzification of data. Neural network control methods are increasingly popular algorithms in many fields based on high-performance and decision-making capabilities [10], but the very large sets of training data required and the resulting computational complexity make them unsuitable for more user-specific applications. Other more novel control schemes have been used in recent research for motion tracking of PAM-driven systems, including the event-triggered neural network tracking control presented in Ref. [11], showing that the field of pneumatic actuator motion control is ongoing and of recent interest. MPC produces optimal predicted control outputs for both current and future inputs. The consideration of future control inputs allows for accurate time-dependent control, and the use of optimisation algorithms in the generation ensures consistently accurate motion. The main drawbacks of MPC are the requirement of an accurate system model and knowledge of future input sequences, but as rehabilitation robots use predetermined sets of motions to provide therapeutic exercises, this second requirement is already fulfilled. As such, this paper will present the development, implementation, and experimental testing of an MPC scheme designed for a platform-based ankle rehabilitation robot driven by four parallel PAMs, as well as an optimisation-based modelling method to account for the requirement of a system model in the controller. Clinical studies have shown that even among patients suffering the same joint-affecting conditions, there is great variety in ankle biomechanics [12,13], further reinforcing the need for compliant and flexible ankle rehabilitation platforms.

Using a dynamic model of the plant, MPC predicts the optimal control input sequence to achieve the desired output. So long as an accurate model of the system can be calculated, this allows for accurate and reliable control of even complex systems, accounting for time variance, position dependence, and other nonlinearities. There are several examples of MPC used in rehabilitation robotics, even aside from systems that use PAMs for actuation [3]. Some examples of walking-based patients following rehabilitation robots using MPC are presented in Refs. [14,15]. In Ref. [14], a robot is designed to automatically follow patients at a set distance, allowing for a walking aid, using MPC to maintain distance and speed. A similar system focused on stroke rehabilitation presented in Ref. [15] uses a weight-bearing support structure to assist lower-limb impaired patients in following predefined paths, using MPC with quadratic regression to accurately track the desired motion. Other examples of MPC used in lower-limb rehabilitation include powered orthoses and exoskeletons, such as those presented in Refs. [16,17]. An impedance controller uses MPC to calculate optimal joint stiffness in a wearable lower-limb rehabilitation exoskeleton developed in Ref. [16]. A position controller for a nonlinear knee exoskeleton using a linearization technique and MPC is developed and compared with several other MPC methods and is presented in Ref. [17]. Upper limb rehabilitation also benefits from the use of MPC, such as the system presented in Ref. [18]. A portable upper limb rehabilitation robot is tested using MPC and PID control for comparison. In fact, PID is often used as a comparison point in control system development for rehabilitation robotics, with results in Refs. [15,17,18] showing comparative data verifying the performance of MPC in their respective applications.

The biggest difficulty in developing MPC is the requirement for a model of the plant. As the predicted control inputs are calculated based on the behaviour of the model, a more accurate model necessarily results in more accurate control. In the case of complex, nonlinear systems, accurate models become difficult to calculate, often resulting in a significant trade-off between accuracy and complexity. PAMs, with the presence of hysteresis, as well as nonlinear input–output relationships and time variance, are notoriously difficult to accurately model. Many different applicable modelling techniques exist for rehabilitation robots, with unique and novel methods present throughout the literature as well. The parallel upper limb rehabilitation robot developed in Ref. [19] uses a kinematics-based model approximating the system to a series of chains, and also discusses the Lagrangian method. An upper limb rehabilitation robot shown in Ref. [20] uses a multi-domain modelling method in SimScape multibody, developed to assist with tracking a patient’s recovery process. In Ref. [21], a fuzzy inference system is used to accurately calculate the forward kinematics of an ankle rehabilitation robot. The fuzzy inference system was optimised using several methods for a comparison of their effectiveness.

Optimisation algorithms are often used for the purpose of dynamic modelling, as they are an ideal choice for calculating the parameters of complex systems without themselves becoming more complicated. PSO is a popular optimisation method, based on its comparatively fast convergence through the usage of a large “swarm” of potential solutions, or particles, which communicate their respective fitness according to the objective function, allowing for informed changing of parameters, resulting in fewer required iterations to reach an optimal solution compared with other optimisation algorithms. An example of a three-degrees-of-freedom (DoF) cylindrical manipulator is presented in Ref. [22], with inverse kinematic identification performed using least squares and recursive least squares, and dynamic parameter identification performed by PSO. With a dynamic model generated using the Lagrange equation, it was found that PSO parameter identification was more accurate than the least squares methods. Another instance of PSO being used comparatively with a least squares identification method is shown in Ref. [23] with a desktop Phantom Omni haptic device. There are many examples of PSO being used for parameter identification in rehabilitation robotics as well. An upper limb rehabilitation device driven by two antagonistic PAM pairs is modelled using a neural network trained using the PSO algorithm in Ref. [24], with results suggesting that the method is suitable for modelling and control of various multi-input multi-output (MIMO) systems. Another upper limb rehabilitation device is shown in Ref. [25] using an advanced PSO algorithm with variable parameters for dynamic modelling. Results show that the proposed method outperforms least squares as well as the basic PSO, with significantly reduced tracking errors and chattering. A full dual lower-limb exoskeleton, shown in Ref. [26], uses a PSO-optimised Support Vector Regression system to identify walking modes in users, and, in experiments with three users, this method is proven to be effective. As is shown in the literature, PSO is a well-documented and suitable algorithm for parameter identification as it converges rapidly to optimal solutions. It has excellent scalability for higher-dimensional search spaces without exponentially increasing complexity or runtime but is also flexible at both the swarm scale and in objective function design to be applied to much smaller optimisation problems too. The flexibility and short time required make it applicable to most parameter identification tasks.

In this paper, a platform-based CARR is used for the development of a novel intelligent control system. The robot is designed to allow for compliant and comfortable rehabilitation of the ankle joint on three rotational DoFs. The aim of this control system is to provide more accurate, stable, and consistent motion of the CARR to make it more suitable for the safe human–robot interaction required when undergoing robot-assisted physical rehabilitation. The actuation method and mechanisms of the CARR create difficulty in accurately modelling and controlling the system by introducing significant nonlinearity and time-dependence. To account for this, a PSO-based modelling method was developed for dynamic parameter identification of PAMs using a phenomenological model template [27]. Using this method, a series of dynamic models will be generated to approximate the behaviour of the actuators in the CARR. These developed models will then be applied to an MPC strategy. Two methodologies will be used: a single-model setup in which each of the four PAMs in the system are assumed to have the same dynamic properties, and a dual-model setup in which the upper and lower pairs will be modelled separately. These single-model and dual-model MPCs will then be experimentally compared, alongside traditional PID control and ILC, to confirm the validity of the proposed method. The CARR has been experimentally validated in multiple experiments including a case study in the treatment of drop-foot [28] and has had multiple different control strategies developed and tested [29,30].

The main contributions are summarised as follows:

1. Real-world application and validation of a computationally efficient algorithm to generate dynamic models of complex, nonlinear systems in the PSO modelling method, initially developed in Ref. [27].
2. Two sets of dynamic models proposed for the CARR in the single-model and dual-model setups.
3. Development of two MPCs using each of the generated model sets.
4. Experimental results and comparative analysis of the performance of these controllers with a traditional PID and ILC, demonstrating the developed MPC’s improved motion accuracy and stability, and justifying its suitability for use in the application of robot-assisted physical rehabilitation of the ankle joint.

This paper starts with a technical description of the CARR’s design and functionality, and an explanation of the kinematic equations used in its control. Subsequently, the PSO modelling method is described followed by the developed MPC system. Then, the methodology for the set of validation experiments is given, followed by results and metrics from these experiments. The findings and important factors are then discussed, and, finally, the work is summarised.

## 2. Robot Design

### 2.1. Layout and Functionality

**Figure 1:**
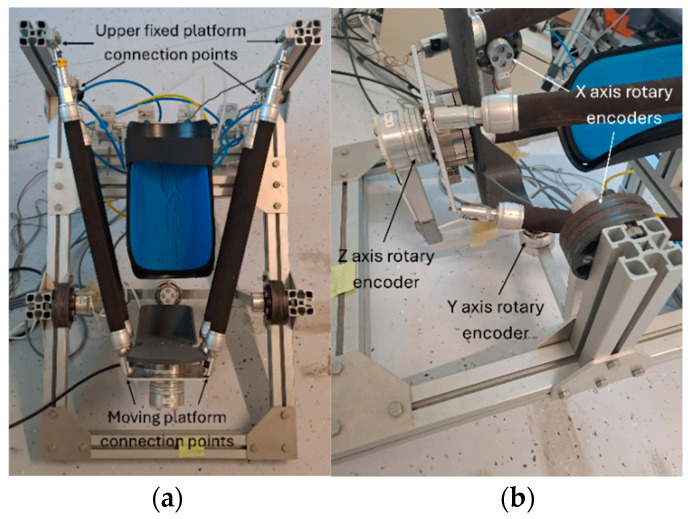
Images of the CARR. (**a**) The PAMs and connection points. (**b**) The bar linkage mechanism for the footplate and the rotary encoders used to measure angular displacement.

**Figure 2:**
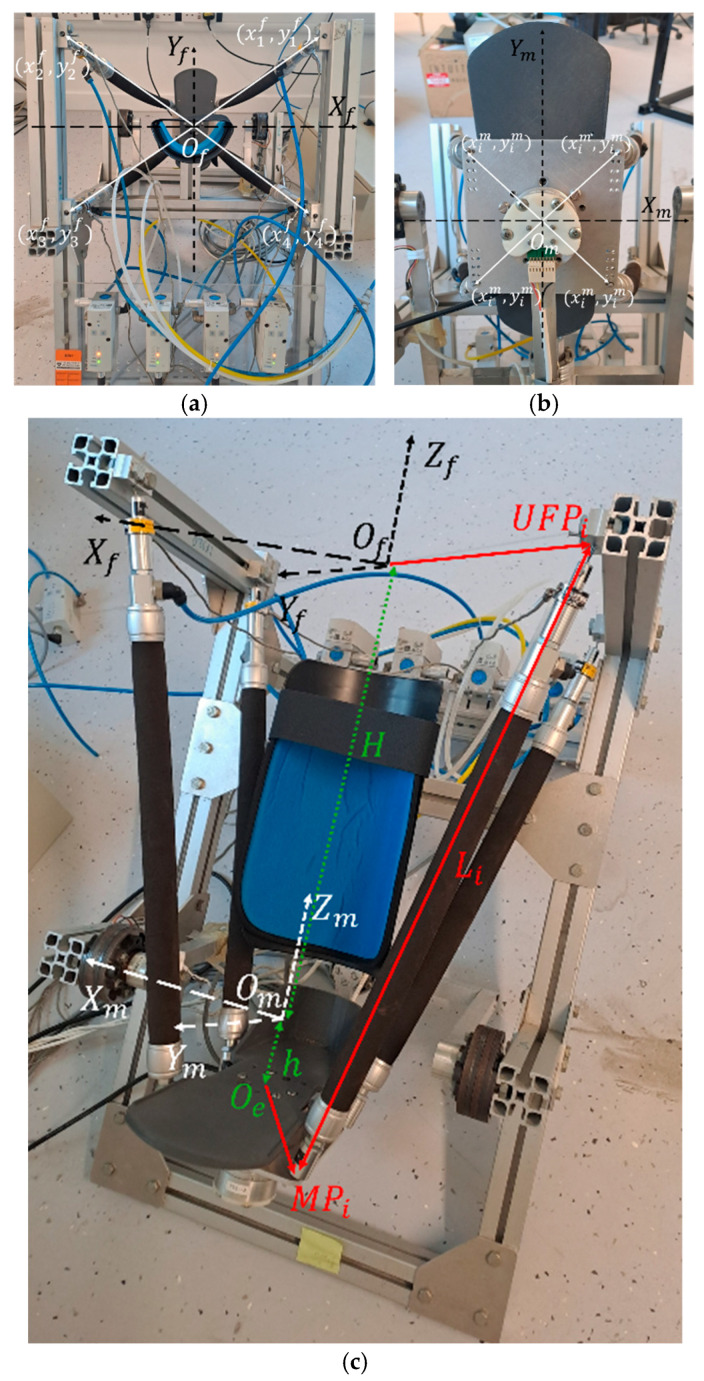
Coordinate system of the CARR. (**a**) shows the upper fixed platform coordinates, (**b**) shows the moving platform coordinates, and (**c**) shows the combined coordinate systems along with the measured values *H*, *h*, and the muscle length $L_{i}$

The CARR is shown in Figure 1 and Figure 2. It consists of two platforms: a fixed platform, the frame, and a mobile one, the footplate. The platforms are connected in two places: by four FESTO DMSP-20-400N PAMs produced by FESTO, based in Esslingen am Neckar, Germany, fixed with one end at points around the footplate and the other at the upper section of the frame, as shown in Figure 1, and via a bar linkage serial mechanism with three rotational DoFs, as shown in Figure 1. This mechanism includes rotary encoders on each of the rotational axis. The PAMs are actuated using four FESTO VPPM-6L-L-1-G18-0L6H proportional pressure regulators. This configuration allows for the CARR to achieve three rotational DoFs, corresponding to the dorsiflexion/plantarflexion, inversion/eversion, and abduction/adduction motions of the ankle joint, by actuating the PAMs in opposed pairs. The use of four PAMs connected at each corner of the footplate end effector and angled outwards in relation to the plane of motion allows for antagonistic force to be applied across the three axes, with the upper and lower pairs acting to operate the X axis, the left- and right-hand pairs acting to operate the Y axis, and diagonally opposed pairs acting to operate the Z axis.

Shown in Figure 2 is the coordinate system used to describe the CARR. The system consists of fixed coordinates describing the fixed platform denoted as $O_{f}X_{f}Y_{f}Z_{f}$ and moving coordinates describing the mobile platform denoted as $O_{m}X_{m}Y_{m}Z_{m}$. The upper connection point on the fixed platform where the actuators are connected is denoted as UFP, while the lower connection point of the fixed platform where the linkage mechanism of the mobile platform is connected is denoted as LFP. The mobile platform consisting of the linkage mechanism and footplate is denoted as MP. The connection point of the ith PAM on the UFP and MP are referred to as ${UFP}_{i}$ and ${MP}_{i}$, respectively. $O_{f}$ refers to the centrepoint of UFP, $O_{m}$ refers to the rotational centre of MP, and $O_{e}$ refers to the centrepoint of the footplate such that it is equidistant from each of ${MP}_{i},\;i=1\;to\;4$. H refers to the distance $\bar{O_{f}O_{m}}$ and *h* refers to the distance $\bar{O_{m}O_{e}}$. These coordinate systems and definitions are displayed graphically in Figure 2 and were originally defined in Ref. [31].

A flaw of the system’s current configuration is that while the actuator arrangement allows for proper control of the X and Y axes in both directions using opposed pairs of PAMs, the methodology for actuation of the Z axis is fundamentally lacking. Using diagonally opposed PAMs at small angles to the footplate results in difficulty accurately controlling the axis due to the force vectors being mostly parallel with the axis of motion. This, combined with the necessary interaction with the other axes while in motion, causes the Z axis of the system to be unstable and inaccurate, a fault not currently rectifiable by software control. Discussion on the potential redesign of the CARR to better control the Z axis will be made towards the end of this paper, but, as this is outside the scope of the study presented here, the experiments will focus on the motion of the X and Y axes for better validation of the developed modelling and control algorithms.

### 2.2. Kinematics

In order for the CARR to be properly modelled and controlled using the methods described in the next section, the length of each PAM must be known throughout online motion. Therefore, the kinematics of the CARR are calculated.

The length of the ith actuator is given by the distance $\bar{{UFP}_{i}{MP}_{i}}$. The coordinates of ${UFP}_{i}$ in relation to $O_{f}$ are described as $\left[x_{i}^{f},\;y_{i}^{f},\;z_{i}^{f}\right]$, and the coordinates of ${MP}_{i}$ in relation to $O_{m}$ are described as $\left[x_{i}^{m},\;y_{i}^{m},\;z_{i}^{m}\right]$. Therefore, the length of the *i*th actuator, denoted as $L_{i}$, can be calculated per Equation (1).

(1) $$L_{i}=\sqrt{{\bar{x_{i}^{f}x_{i}^{m}}}^{2}+{\bar{y_{i}^{f}y_{i}^{m}}}^{2}+{\bar{z_{i}^{f}z_{i}^{m}}}^{2}}$$

These cartesian distances can be calculated using the angular displacement measured by the rotary encoders on the linkage mechanism of the mobile platform, $\theta_{x}$, $\theta_{y}$, and $\theta_{z}$, as defined in Equations (2)–(4).

(2) $$\bar{x_{i}^{f}x_{i}^{m}}=x_{i}^{f}+h\left(\sin\left(\theta_{x}\right)\sin\left(\theta_{z}\right)+\cos\left(\theta_{x}\right)\sin\left(\theta_{y}\right)\cos\left(\theta_{z}\right)\right)+y_{i}^{m}\left(\cos\left(\theta_{x}\right)\sin\left(\theta_{z}\right)-\sin\left(\theta_{x}\right)\sin\left(\theta_{y}\right)\cos\left(\theta_{z}\right)\right)-x_{i}^{m}\cos\left(\theta_{y}\right)\cos\left(\theta_{z}\right)$$

(3) $$\bar{y_{i}^{f}y_{i}^{m}}=-y_{i}^{f}+h\left(\sin\left(\theta_{x}\right)\cos\left(\theta_{z}\right)-\cos\left(\theta_{x}\right)\sin\left(\theta_{y}\right)\sin\left(\theta_{z}\right)\right)+y_{i}^{m}\left(\cos\left(\theta_{x}\right)cos\left(\theta_{z}\right)+\sin\left(\theta_{x}\right)\sin\left(\theta_{y}\right)\sin\left(\theta_{z}\right)\right)+x_{i}^{m}\cos\left(\theta_{y}\right)\sin\left(\theta_{z}\right)$$

(4) $$\bar{z_{i}^{f}z_{i}^{m}}=H+x_{i}^{m}\sin\left(\theta_{y}\right)+h\cos\left(\theta_{x}\right)\cos\left(\theta_{y}\right)-y_{i}^{m}\sin\left(\theta_{x}\right)\cos\left(\theta_{y}\right)$$

The measured values of the coordinates of each of ${UFP}_{i}$ and ${MP}_{i}$ are shown in Table 1. The values of *H* and *h* are H=415 and h=120. All values are measured in millimetres relative to the centrepoints $O_{f}$ and $O_{m}$.

The outputs calculated from Equation (1) were scaled to the range [0–10] to allow for simpler calculations in the modelling and control stages.

## 3. Modelling and Control

### 3.1. Modelling

**Figure 3:**
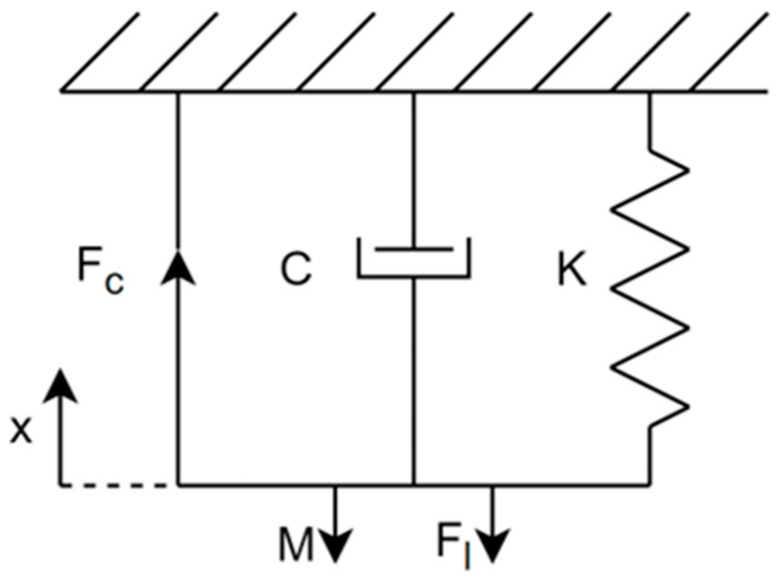
Phenomenological model approximation of the PAM.

The PSO-based dynamic modelling method developed in Ref. [27] was adapted and used to calculate model parameters for the PAMs used in actuating the CARR. The modelling algorithm relies on a phenomenological model approximation of the PAM as a mass–spring–damper system, with the equation of motion shown in Equation (5).

(5) $$M\ddot{x}\left(t\right)+D\dot{x}\left(t\right)+Cx\left(t\right)=F_{c}-F_{l}$$

where x(t) is the displacement of the actuator from the nominal position at time step *t*, *M* is the mass of the actuator, *D* is the damping constant, *C* is the spring constant, $F_{c}$ is the contractile force applied by the internal pressure of the PAM, and $F_{l}$ is the external load applied, effectively acting as a negating force value to $F_{c}$. In the case of the unloaded CARR, $F_{l}$ would represent the weight of the mobile platform linkage mechanism, the footplate, and any friction acting at the rotational joints of the system. In the case of a loaded CARR, this value would increase based on forces applied by a user and would differ on a case-by-case basis. The model is shown in Figure 3.

Based on this model, the state-space representation of the system of a single PAM can be written as per Equations (6) and (7).

(6) $$\dot{X}\left(t\right)=\left[\begin{array}{c} {\dot{x}}_{1}(t)\\ {\dot{x}}_{2}(t) \end{array}\right]=\left[\begin{array}{cc} 0 & 1\\ -\frac{K}{M} & -\frac{C}{M} \end{array}\right]\left[\begin{array}{c} x_{1}(t)\\ x_{2}(t) \end{array}\right]+\left[\begin{array}{c} 0\\ \frac{1}{M} \end{array}\right]U(t)$$

(7) $$y\left(t\right)=\left[\begin{array}{cc} 1 & 0 \end{array}\right]\left[\begin{array}{c} x_{1}(t)\\ x_{2}(t) \end{array}\right]+\left[0\right]U\left(t\right)$$

where $x_{1}$ and $x_{2}$ are state variables such that $x_{1}\left(t\right)=x\left(t\right)$ and $x_{2}\left(t\right)=\dot{x}(t)$ and *U* is the system input.

Given the three unknown values in this representation, $-\frac{K}{M}$, $-\frac{C}{M}$, and $\frac{1}{M}$, a PSO algorithm can be used to calculate their values. Henceforth, these values will be referred to as $P_{1}$, $P_{2}$, and $P_{3}$, respectively. The equation for each model is therefore given by Equation (8).

(8) $$\dot{X}\left(t\right)=\left[\begin{array}{c} {\dot{x}}_{1}(t)\\ {\dot{x}}_{2}(t) \end{array}\right]=\left[\begin{array}{cc} 0 & 1\\ P_{1} & P_{2} \end{array}\right]\left[\begin{array}{c} x_{1}(t)\\ x_{2}(t) \end{array}\right]+\left[\begin{array}{c} 0\\ P_{3} \end{array}\right]U(t)$$

The algorithm is based on a swarm of particles in an N-dimensional search space, with N being the number of parameters, in this case three, with each particle’s position in the search space represented by its set of parameters. At each time step, each particle’s position is updated according to Equations (9) and (10).

(9) $$V_{i}(k+1)=WV_{i}(k)+C1Rand(X_{i}best(k)-X_{i}(k))+C2Rand(Gbest\left(k\right)-X_{i}\left(k\right))$$

(10) $$X_{i}\left(k+1\right)=X_{i}\left(k\right)+V_{i}(k+1)$$

where *i* is the current particle; *k* is the current iteration of the algorithm; *V* is the particle’s velocity in the search space; X is a particle’s position in the search space quantified by its parameters; Rand is a random value between 0 and 1; $X_{i}best$ is the position of the current particle which achieved the best (lowest) fitness; Gbest is the global best position of all particles in the swarm; and W, C1, and C2 are algorithm parameters that are tuned manually.

Given a comparison point generated by applying a predetermined sequence of inputs to the plant, the fitness of each particle is calculated using the error value between the behaviour of the phenomenological model, using that particle’s parameters in place of the unknown values, and the behaviour of the plant. A sin wave input with amplitude 2 and frequency 0.05 Hz and repeated twice was used as the input to the upper pair of PAMs, with the same waveform inverted and used as the input for the lower pair. This results in a constant and smooth sinusoidal changing of the X angle of the system. The values for the amplitude and frequency were tuned manually based on the requirements of the system in a rehabilitation setting to best model for the intended application, keeping range, speed, and acceleration of motion within expected operational limits. Using the measured output of the CARR joint angles, the inverse kinematic equations of the system were used to calculate the length of each PAM. The resulting length measurements were used as setpoints to calculate the root mean squared error (RMSE) of each particle. These values were used as the fitness function for the particles in the PSO algorithm.

It is recognised that PAMs are highly nonlinear actuators, particularly towards the extremes of allowable motion. PAMs possess uniquely nonlinear properties, including hysteresis due to friction in the rubber casing and displacement- and time-variant motion responses due to the compressibility and changing flow rates of the pressurised air used in their actuation. The simplification of these actuators to a three-element approximation may not account for all potential nonlinearities, and while possible methods to address these are presented in Ref. [27] these resulted in other inaccuracies when using the models alongside other algorithms such as MPC. When used in combination with one another, as well as when actuating a heavier structure such as the mobile platform present on the CARR, the changes in motion response due to nonlinearities in the PAMs have a much smaller impact on accuracy than when used individually. Therefore, the simplification to a mass–spring–damper system was deemed appropriate for this application, especially considering the greatly increased complexity required to account for nonlinearities, resulting in a relatively small potential improvement to accuracy.

Two separate modelling setups were used for comparative experiments, a single-model setup and a dual-model setup. In the single-model setup, the four PAMs used in the CARR were assumed to have the same dynamic properties and a single set of three parameters were calculated using the PSO method, forming a single dynamic model which was used to model all four PAMs. The fitness of each particle was calculated by summing the RMSE calculated for each of the four PAM setpoint motions. In the dual-model setup, the upper pair of actuators was assumed to have different properties to the lower pair based on their angle to the ground and position in the structure of the CARR, so two separate sets of parameters were calculated using separate PSO algorithms running alongside each other, one summing the RMSE values for the upper two PAMs to calculate fitness values and the other summing the RMSE values for the lower two to calculate fitness. Using this setup, two independent models were generated for each pair of PAMs. The parameters used for the PSO algorithm were W = 0.9 to prevent divergent learning of the swarm, C1 = C2 = 2 to increase the optimisation rate and reduce the number of iterations required to reach an optimal solution, a swarm size of 50 as a trade-off between the optimisation rate and computational complexity, and each model setup was run for 20 iterations as this was the point by which local optimal solutions were most often reached. The parameters for the models generated for these experiments to be used in Equation (8) for the state-space representation of the models are shown in Table 2.

### 3.2. Control

Using the models generated by the proposed PSO modelling method, an MPC strategy can be created for position control of the CARR. MPC uses an optimisation algorithm, together with prior knowledge of the intended input sequence and a model of the plant, to predict an optimal control signal. At each time period t, an optimisation function calculates a cost-minimising control output for the defined prediction period T based on the input sequence for that period and the predicted behaviour of the plant from the model. The first stage of this control output is applied to the system, and the rest is discarded for the process to be repeated at the next time step. MPC is therefore suitable for application in nonlinear systems due to its predictive nature, as well as for unusual or time-variant input sequences so long as they are known beforehand. The use of the PSO modelling method also allows this implementation of MPC to accurately control complex systems while remaining computationally efficient.

The optimisation function used in this implementation of MPC is linear quadratic programming. The cost function of this is given by Equation (11).

(11) $$J=\sum_{t}^{T}q{\left(y-s\right)}^{2}+\sum_{t}^{T}r∆u^{2}$$

where *t* is the current time step, *T* is the prediction period, *q* is the output error weight parameter, *y* is the predicted system output, *s* is the setpoint, *r* is the control weight parameter, and ∆u is the predicted change in the control value such that $∆u=u_{k}-u_{k-1}$, with $u_{k}$ being the control value taken at time step *k*.

This cost function was chosen to allow both the predicted error and predicted control values to influence the decision in optimal control output. Both the error value and control change are squared to keep the value of J positive and prevent them from counteracting each other, and the resulting quadratic function is best optimised using quadratic programming. For the MPC used in this study, q=1 and r=20. These values were chosen to ensure that predicted error and predicted control value change are both properly accounted for based on their normalised values during runtime. The prediction period was chosen as *T* = 10. This value was chosen to allow sufficient time steps to be considered in the optimisation algorithm to give an accurate optimal solution, but as a greater prediction period reduces the efficiency of the algorithm, a sufficiently small value was needed to keep unnecessary calculations to a minimum.

Based on this cost function, optimal control output values are calculated by solving Equation (12) for *u*.

(12) $$\frac{\delta J}{\delta u}=0$$

**Figure 4:**
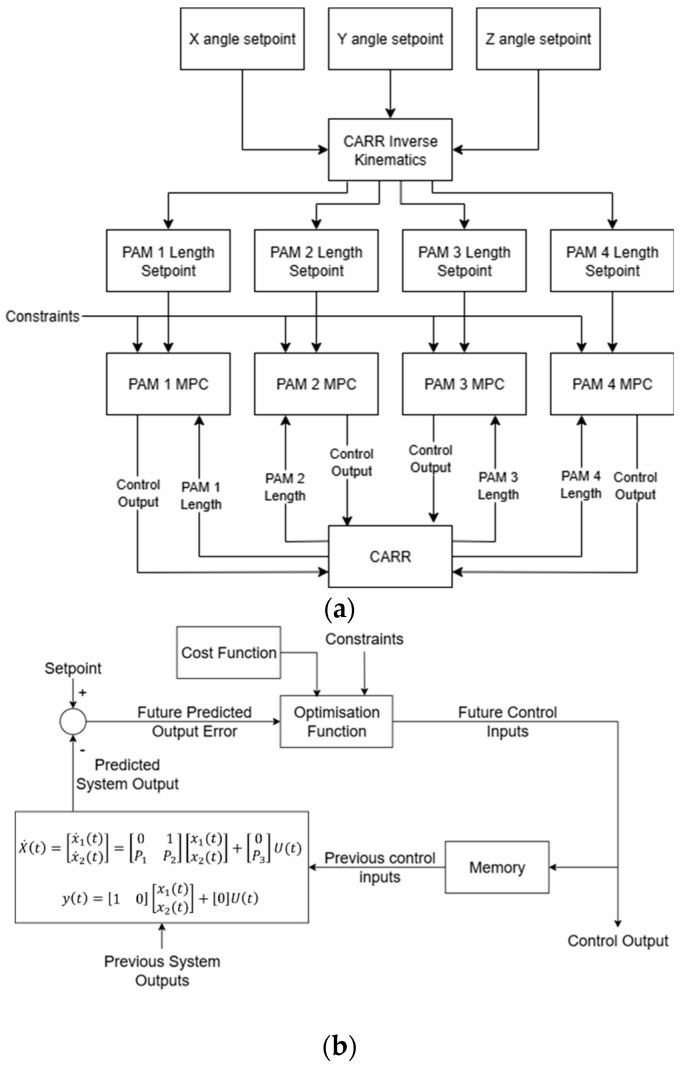
Flowcharts showing the control structure for the CARR, including setpoint calculation and structure of the MPC loop including application of the PSO dynamic model. (**a**) shows a diagram of the MPC for the four PAMs and the overall control structure for the CARR; (**b**) shows the structure of the PAM MPC blocks.

The models generated by the PSO method, as detailed earlier, describe the dynamics of the individual PAMs rather than the CARR as a whole. Therefore, four separate MPCs were used for controlling the system. While a MIMO or centralised control algorithm accounting for the CARR as a combined system would allow for cooperative action of the PAMs and may improve accuracy, this would also necessitate a far more complex model of the CARR than is required of the PAMs individually. It may also introduce further nonlinearities and time variance to the MPC and would make relative force output readings more complicated. This potential alternate control architecture is therefore left as future work in the case of this study. The input of the system was defined as the desired angles on the X, Y and Z axes for the footplate. While the Z axis will not be implemented as part of the controller’s validation due to instability, as previously discussed, it is a necessary measure for the kinematic equations of the system and must therefore be included in the control architecture, so all input values for the Z axis will be set to 0 for the purposes of this study. Input sequences for each actuator were calculated using the inverse kinematic equations of the system. Figure 4 shows a diagram of the MPC for the four PAMs and the overall control structure for the CARR.

For an experimental comparison of the developed MPC strategies, a traditional PID controller and ILC were developed for the CARR. Similarly to the functioning of the MPC, a separate controller was used for each of the four PAMs in all cases, and the setpoint length for each was calculated using the inverse kinematics of the system.

As neither PID control nor ILC require that the entire input sequence be known by the controller beforehand, the setpoints for each PAM were calculated online during motion. The parameters of the PID controller used in this study were $K_{c}=20$, $T_{i}=0.009$, and $T_{d}=0.00225$. These values were tuned manually for fast response to the setpoint while minimising instability.

The ILC used in this study was a classical implementation. The control output of the algorithm is given in Equation (13).

(13) $$u\left(t\right)=u_{old}\left(t\right)+pe_{old}(t)$$

where $u\left(t\right)$ is the control output, $u_{old}\left(t\right)$ is the control output of the previous iteration, $e_{old}(t)$ is the measured error during the previous iteration, and *p* is the learning rate which was set to 20, again tuned manually for fast convergence to the setpoint in a low number of learning periods while minimising instability, overfitting, and divergent behaviour. The length of each learning iteration was a single wavelength of the input for each experiment.

## 4. Experiment Methodology

Each experiment uses a set waveform setpoint for the X axis, Y axis or both axes of the CARR. The instability of the motion of the Z axis due to the mechanical shortcomings of the CARR means that any experimental data gathered would not be reliable or easily compared and will not allow for proper validation of the developed controllers, which is the goal of this study. Therefore, Z axis motion will not be implemented as part of these experiments, and all values input to the kinematic equations for the Z axis will be set to 0.

Table 3 shows the parameters for each waveform in each experiment. Amplitude values are normalised based on the motion range of the CARR on each of the X and Y axes and the direct voltage outputs from the angular encoders.

For each experiment, each of the single-model MPC, dual-model MPC, PID and ILC controllers will have the same unaltered waveform input as the setpoint for each axis, allowing for comparable results. For the MPC and PID algorithms, each experiment will be run for 5 waveforms, except in the cases of the rapid motion experiments where they will be run for 10 waveforms. As the first wavelength of each experiment for the ILC is only learning and no control output can be calculated, each experiment will be run for 6 waveforms with the first omitted from the results, or for 12 waveforms in the case of rapid motion with the first 2 omitted.

## 5. Results

**Figure 5:**
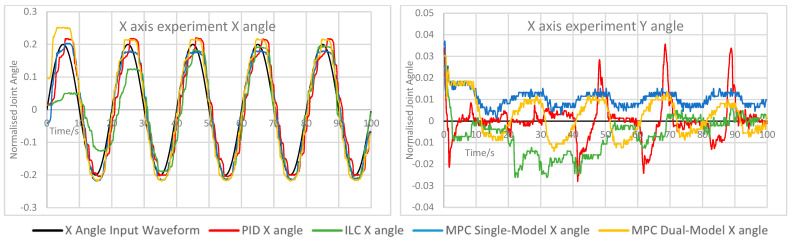
Measured angle values for the X and Y axes of the CARR during the X axis experiment.

**Figure 6:**
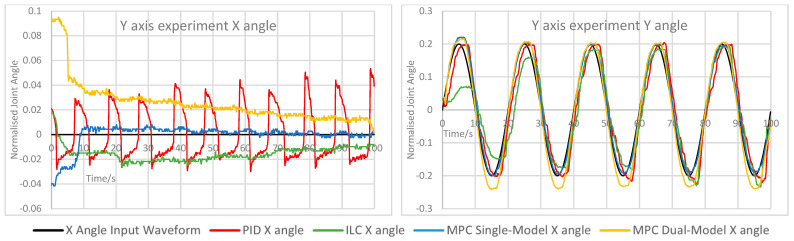
Measured angle values for the X and Y axes of the CARR during the Y axis experiment.

**Figure 7:**
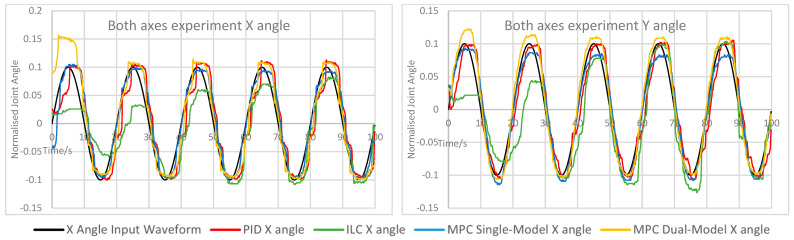
Measured angle values for the X and Y axes of the CARR during the X and Y axes experiment.

**Figure 8:**
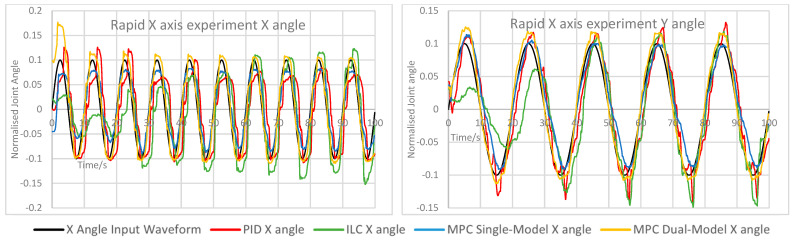
Measured angle values for the X and Y axes of the CARR during the rapid X axis experiment.

**Figure 9:**
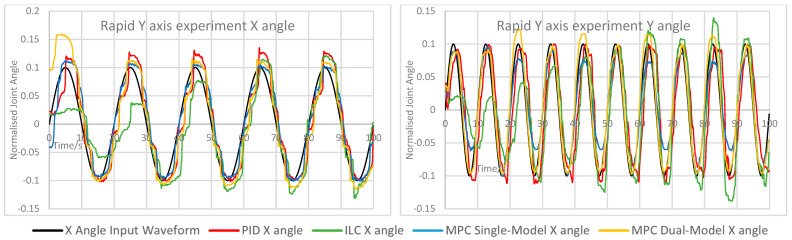
Measured angle values for the X and Y axes of the CARR during the rapid Y axis experiment.

The measured X and Y angles for each experiment under each control scheme were measured and overlaid for comparison. The angle error for each motion was calculated and overlaid to demonstrate the relative performance of each controller. The measured angle results for each experiment are shown here in Figure 5, Figure 6, Figure 7, Figure 8 and Figure 9. Key comparison metrics were calculated and are shown in Table 4.

## 6. Discussion

The experiments performed in this study were designed to approximate the requirements of the CARR in a rehabilitation setting. The frequency and amplitude of the input waveforms were selected based on the speed and angle of exercise requirements during ankle rehabilitation. The rapid motion experiments used frequency values on the extreme upper end of the required values in rehabilitation, which usually uses slow, measured motions. This was primarily a test of the capability of the controllers and the CARR to perform well during faster motion, and to determine the system’s ability to perform motion simultaneously on two axes with different speeds. In experiments where both the X and Y angles were in motion, the chosen amplitude of motion was smaller, as the CARR exhibits some accuracy issues at the extremes of motion while the footplate is undergoing multiple angle motions simultaneously. The use of a MIMO model for the CARR and an associated MIMO control system could address this issue, as well as mitigate some of the performance issues found in some controllers while multiple axes were in motion at once. In order for each controller to perform as well as possible, some recalibration of the rotary encoder on the X axis was required, which resulted in some temporary inaccuracy at the immediate start of each experiment on this axis, especially noticeable in the two MPC implementations. Specific performance metrics of the RMSE, peak error, and overshoot percentage values are listed for both axes in Table 4 and will be referenced throughout this discussion.

In general, the performance of the PID controller was the worst of the controllers, with a higher RMSE and peak error in the majority of experiments than the MPC implementations, and the least consistent motion of all controllers. Where an axis was expected to remain at 0 angular displacement, the peak error under the PID was invariably the highest and significant motion was observed. In several examples, namely the Y axis experiment and both rapid motion experiments, the percentage overshoot of the Y axis under PID control was significant, and in the rapid motion experiment the X axis suffered both over- and undershooting at different points. In terms of smoothness of motion, an important metric for safety and comfort in rehabilitation exercises and often regarded as a more important measure of success in such a device than motion tracking, all control schemes showed some evidence of chattering. This is likely due to the construction of the CARR and the inherent friction present in the actuators and joints on the footplate mechanism. However, the chattering was most significant under PID control, and in many cases the motion would briefly change direction, most noticeably on the X axis when both the X and Y axes were in motion.

As to be expected, ILC began each experiment with poor accuracy and improved over the course of the waveform, with each wavelength reducing the tracking error in most cases. This includes cases where an axis was expected to remain at 0, and in the case of the X axis, resulted in small peak error and RMSE values. However, there is evidence of overfitting in some experiments, mostly during rapid motion, where overshooting of the setpoint becomes more common and significant towards the end of the waveform, as indicated by the very high overshoot percentage values, suggesting instability. In terms of motion smoothness, the ILC had similar but less severe chattering than the PID, with fewer examples of fully changing the direction of motion.

Both MPC implementations performed similarly; however, there were some defining features of both that caused them to outperform each other in certain scenarios. The single-model MPC converged quickly to 0 under experiments where an axis was to remain at 0 and maintained a steady value consistently, as evidenced by the lowest RMSE and peak error values on the X axis and comparably low values for the Y axis. Its percentage overshoot values were the lowest of all controllers in almost every case by a significant margin; however, it did show the only cases of a negative overshoot percentage, indicating that while both axes are in motion, the maximum rotational values were never fully reached. The chattering effect under single-model MPC is lower than under PID control and ILC, resulting in smoother motion.

The dual-model MPC, while having generally similar behaviour to the single-model MPC, did show the closest motion to the setpoint under rapid motion, with the lowest RMSE values of all controllers, as well as either the lowest or nearly the lowest RMSE and peak error for the Y axis in every experiment. Chattering behaviour was best accounted for under this control scheme, with very little evidence of this unwanted motion in all experiments other than on the X axis while both the X and Y axes were in motion. The main drawback of the dual-model MPC was overshooting, which was present in every experiment and noted in the percentage overshoot values; however, unlike the overshooting present under the PID and in some cases under ILC, this motion remained smooth and consistent. A possible cause for this overshooting is that the PAM models become less accurate at the extremes of motion due to dynamic nonlinearity, therefore causing the predicted optimal control values to be less accurate towards the greatest displacement values. This would also explain the undershooting seen in the single-model MPC, and could therefore possibly be rectified by more accurate models or more complex ones which consider the time-varying nature of the actuators.

MPC has proven to be significantly more effective than traditional control like the PID for nonlinear, complex systems like the CARR, and is capable of more reliable accurate motion in more specific use cases than ILC. However, the fact that both MPC implementations have drawbacks suggests that there is still work to be done in developing robust controllers.

## 7. Conclusions

In this study, an ankle rehabilitation robot actuated by four antagonistic PAMs, the CARR, was used as the platform for development and testing of an intelligence-based control system. A PSO-based dynamic modelling method and two MPC schemes were developed for more accurate and smooth motion of the device in a rehabilitation setting. The modelling method, initially developed in Ref. [27], was adapted for use in the MIMO system, and uses a phenomenological model template of PAMs and the PSO algorithm to calculate computationally efficient, yet accurate, models of the nonlinear actuator. Two different setups were used in both the modelling and control of the CARR: a single-model setup in which each of the four PAMs in the robot were assumed to have the same dynamic properties and were therefore modelled with the same parameters, and a dual-model setup in which the top pair of PAMs were assumed to have different dynamic properties to the bottom pair, resulting in two different models.

These models were then used in MPC schemes in experiments to test for their motion tracking accuracy and motion smoothness with the CARR. A traditional PID controller and ILC were also used as comparison points to determine the relative increase in the effectiveness of these MPC algorithms. An experiment scheme was devised using standard sin wave input setpoints for the angular displacement of the X and Y axes of the CARR, and each of these setpoints were used in the PID controller, ILC, and both the single-model and dual-model MPC setups to allow for a comparison of their performance. The experiments included actuating the two axes separately, as well as simultaneously and at different speeds, to test a range of applications. It was found that the MPC algorithms both outperformed PID in terms of absolute error values, as well as smoothness of motion, as did ILC after sufficient learning periods. PID control exhibited the worst chattering effect and either over- or undershot the setpoint in most cases. ILC could converge to accurate and smooth motion but showed some evidence of overfitting towards the end of experiments involving both axes and especially under rapid motion. The single-model MPC had the best behaviour when only a single axis was in motion at once, keeping the unmoving axis consistently stable. However, it suffered from a decrease in accuracy at higher-frequency motion, as well as when both axes were moving simultaneously. The dual-model MPC had the best performance for the Y axis and better accuracy while both axes were in motion. The dual-model MPC had the least evidence of chattering, only apparent on the X axis with both axes in motion, resulting in the smoothest tracking results. It did show evidence of overshooting, although not as significant or sharp as under PID control. The difference in performance between the controllers, and the generally lower accuracy and ROM achieved by the system as a whole while multiple axes were in motion at once, could be improved with the use of a MIMO approach to the system, as the relative interactions between each PAM and each of the three axes are not wholly addressed with the current setup. The reduction in sharp, unwanted motion, like chattering, and motion outside of the desired trajectory, like overshooting, is important for patient safety and comfort. Exercise trajectories must be gentle to keep patients comfortable and help them engage with the activity, and the extremes of motion are carefully considered to not overextend the joint. Inherent compliance added by flexible actuators can assist with this, but it is also important that control strategies minimise their impact as well.

The primary successes of the developed MPCs over other controllers are stability, with lower chattering than the PID and no divergent learning as with ILC, and the ability to respond well to complex MIMO situations with both axes of the CARR moving at once and at different speeds. These successes prove the validity and accuracy of the proposed PSO-generated models for complex MIMO systems as well. As such, based on their accuracy and motion smoothness in the experiments presented here, both the PSO-based modelling method and the resulting MPC schemes are suitable for further research in clinical trials with the CARR. The relative successes of the single-model and dual-model MPCs can be used to the system’s advantage with the addition of a switching-mode or adaptive controller, allowing the shortcomings and benefits to counteract each other in certain cases where one may be preferred over the other. The robustness and response to external disturbance of the developed controllers will be further validated in future work in online experiments with participants to ensure that the system is appropriate and suitable for its intended use, in rehabilitation of the ankle joint.

The CARR itself will require a mechanical redesign to rectify the issues with Z axis motion, as the current PAM configuration makes this axis unstable and inaccurate. This could be done with the addition of a rotary actuator on the footplate to assist with motion, or repositioning of the PAMs to better apply force in the required direction.

## Figures and Tables

**Table 1:** Coordinates of PAM connection points.

Actuator Affixed Point	$x_{i}$	$y_{i}$	$z_{i}$
${UFP}_{1}$	202.5	140	0
${UFP}_{2}$	−202.5	140	0
${UFP}_{3}$	−202.5	−140	0
${UFP}_{4}$	202.5	−140	0
${MP}_{1}$	65	60	−120
${MP}_{2}$	−65	60	−120
${MP}_{3}$	−65	−60	−120
${MP}_{4}$	65	−60	−120

**Table 2:** Model parameters.

Model Setup	$P_{1}$	$P_{2}$	$P_{3}$
Single-Model Setup	0.547499	0.124483	0.445087
Dual-Model Setup—Upper PAMs	0.778931	0.032553	0.387574
Dual-Model Setup—Lower PAMs	0.134703	0.595939	0.335659

**Table 3:** Setpoint waveform parameters.

Experiment	X Axis Frequency/Hz	X Axis Amplitude	Y Axis Frequency/Hz	Y Axis Amplitude
X axis	0.05	0.2	0	0
Y axis	0	0	0.05	0.2
X and Y axes	0.05	0.1	0.05	0.1
Rapid X axis	0.1	0.1	0.05	0.1
Rapid Y axis	0.05	0.1	1	0.1

**Table 4:** Result metrics.

Experiment	Controller	RMSE		Peak Error		Percentage Overshoot	
		X Axis	Y Axis	X Axis	Y Axis	X Axis	Y Axis
X axis	PID	0.0485	0.0088	0.1081	0.0356	10.4	N/A
	ILC	0.0598	0.0109	0.1309	0.0261	8.2	N/A
	MPC Single-Model	0.0294	0.0112	0.0654	0.0151	8.6	N/A
	MPC Dual-Model	0.0366	0.0092	0.0728	0.0140	9.1	N/A
Y axis	PID	0.0211	0.0461	0.0534	0.1077	N/A	13.5
	ILC	0.0166	0.0527	0.0255	0.1349	N/A	16.7
	MPC Single-Model	0.0092	0.0182	0.0080	0.0459	N/A	2.7
	MPC Dual-Model	0.0308	0.0275	0.0307	0.0565	N/A	20.1
X and Y axes	PID	0.0301	0.0214	0.0614	0.0542	12.9	6.9
	ILC	0.0370	0.0354	0.0947	0.1076	8.4	26.4
	MPC Single-Model	0.0192	0.0142	0.0431	0.0286	−0.3	9.4
	MPC Dual-Model	0.0270	0.0152	0.0670	0.0363	14.8	13.7
Rapid X axis	PID	0.0553	0.2545	0.1148	0.0541	22.8	41.3
	ILC	0.0554	0.0389	0.1408	0.1023	51.9	48.8
	MPC Single-Model	0.0248	0.0213	0.0436	0.0462	−10.7	5.1
	MPC Dual-Model	0.0230	0.0131	0.0767	0.0249	18.2	18.6
Rapid Y axis	PID	0.0277	0.0440	0.0571	0.1058	35.2	11.9
	ILC	0.0407	0.0481	0.1049	0.1213	31.7	39.8
	MPC Single-Model	0.0217	0.0199	0.0459	0.0463	6.6	−1.1
	MPC Dual-Model	0.0306	0.0129	0.0669	0.0331	15.8	22.2

## Data Availability

The data presented in this study are available on request from the corresponding author.
